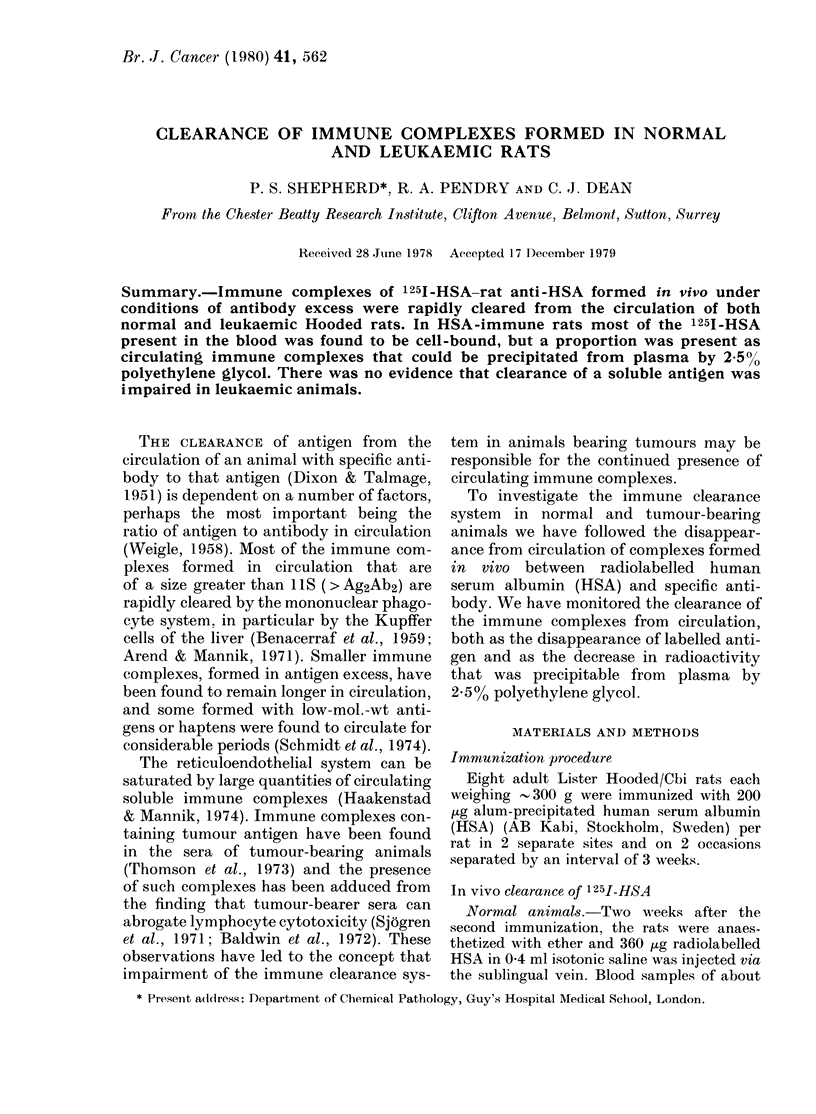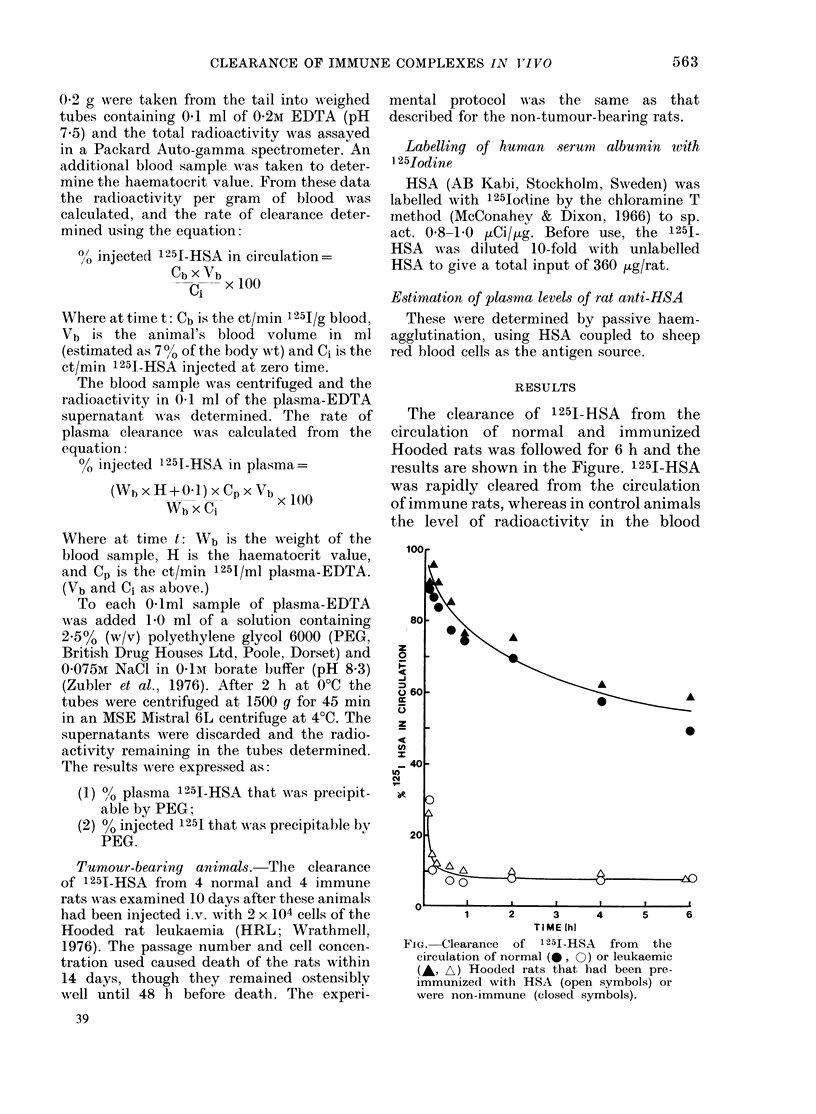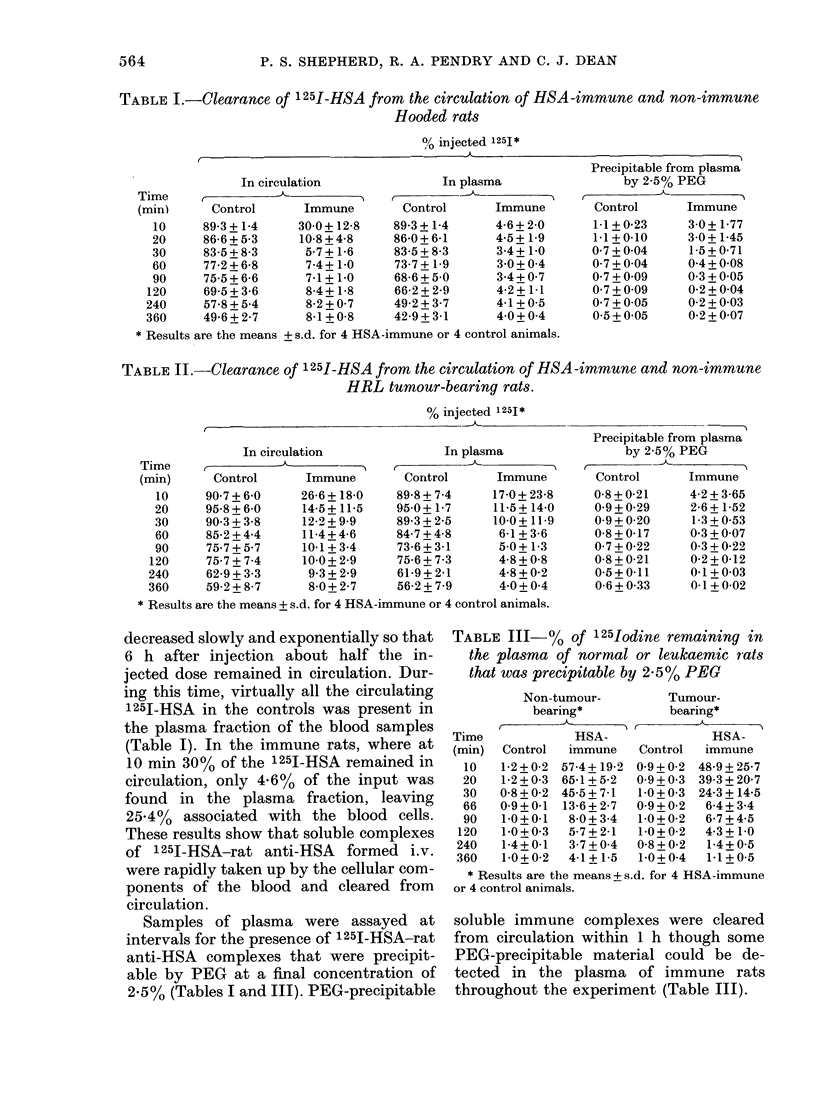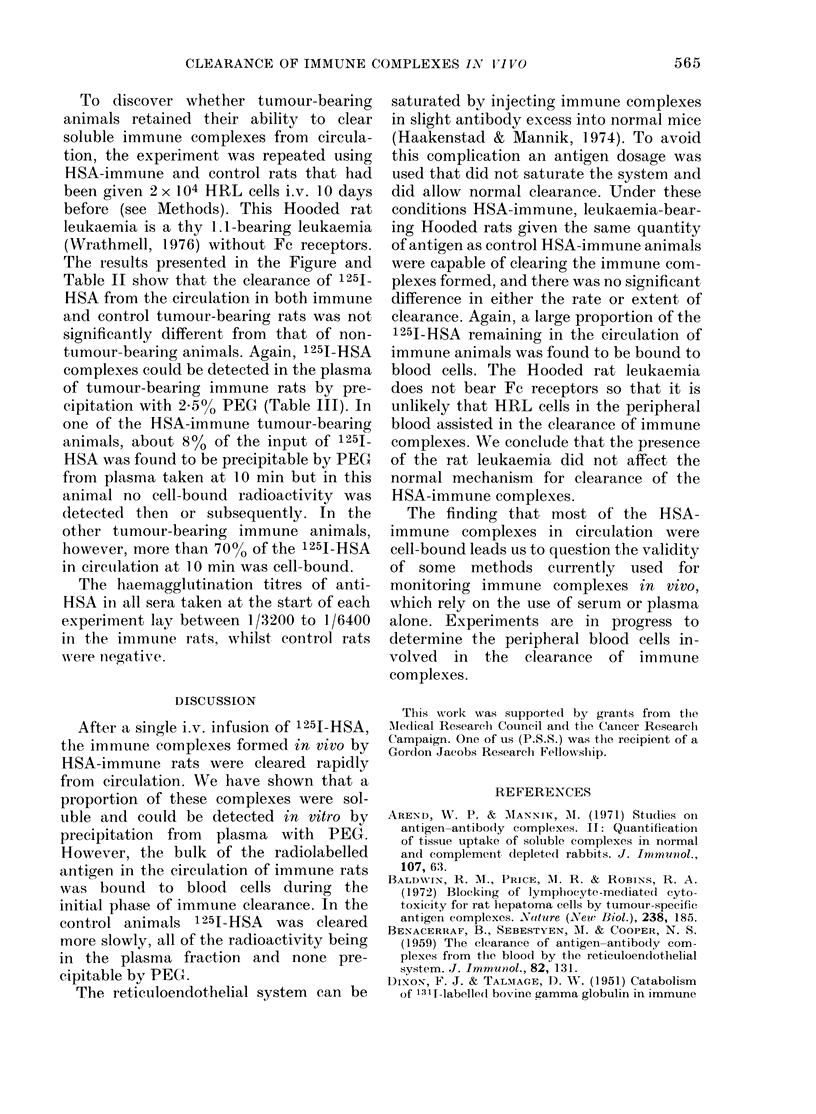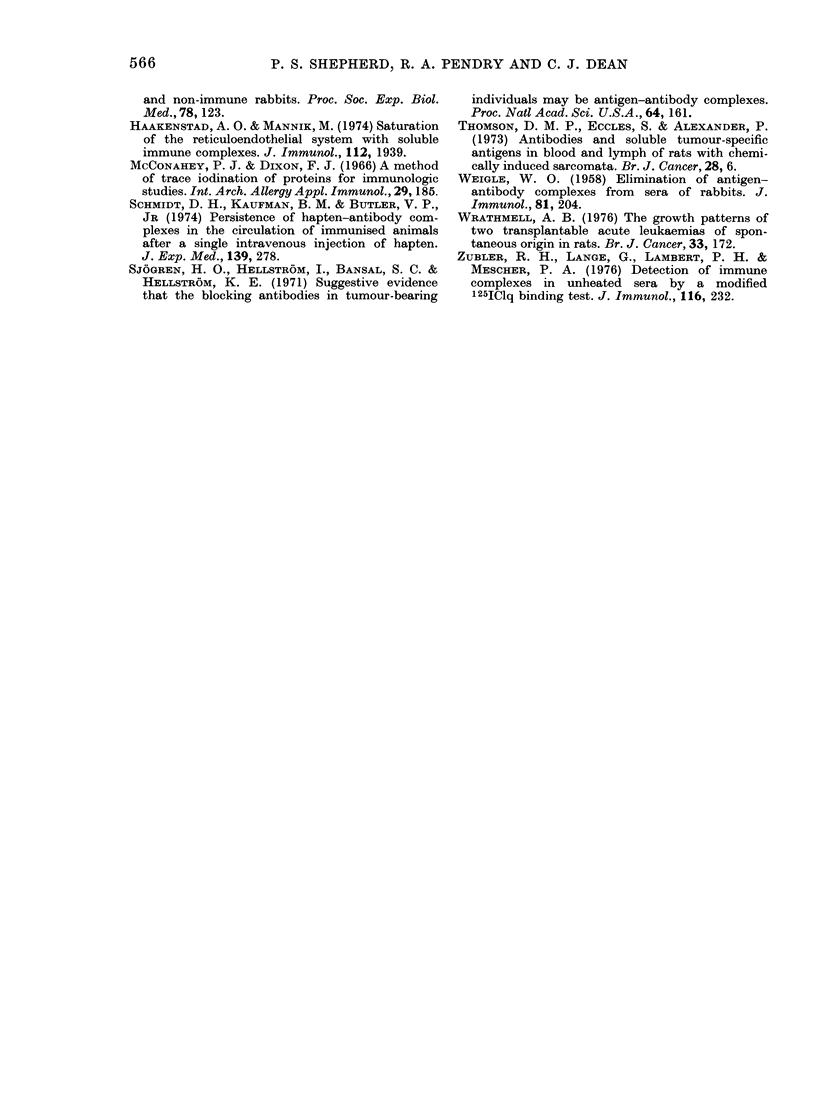# Clearance of immune complexes formed in normal and leukaemic rats.

**DOI:** 10.1038/bjc.1980.98

**Published:** 1980-04

**Authors:** P. S. Shepherd, R. A. Pendry, C. J. Dean

## Abstract

Immune complexes of 125I-HSA-rat anti-HSA formed in vivo under conditions of antibody excess were rapidly cleared from the circulation of both normal and leukaemic Hooded rats. In HSA-immune rats most of the 125I-HSA present in the blood was found to be cell-bound, but a proportion was present as circulating immune complexes that could be precipitated from plasma by 2.5% polyethylene glycol. There was no evidence that clearance of a soluble antigen was impaired in leukaemic animals.


*Br. J. Cancer* (1980) **41**, 562

### CLEARANCE OF IMMUNE COMPLEXES FORMED IN NORMAL AND LEUKAEMIC RATS

P. S. SHEPHERD*, R. A. PENDRY AND C. J. DEAN

*From the Chester Beatty Research Institute, Clifton Avenue, Belmont, Sutton, Surrey*

Received 28 June 1978 Accepted 17 December 1979

**Summary.—Immune complexes of $^{125}$I-HSA–rat anti-HSA formed *in vivo* under conditions of antibody excess were rapidly cleared from the circulation of both normal and leukaemic Hooded rats. In HSA-immune rats most of the $^{125}$I-HSA present in the blood was found to be cell-bound, but a proportion was present as circulating immune complexes that could be precipitated from plasma by 2·5% polyethylene glycol. There was no evidence that clearance of a soluble antigen was impaired in leukaemic animals.**

THE CLEARANCE of antigen from the circulation of an animal with specific antibody to that antigen (Dixon & Talmage, 1951) is dependent on a number of factors, perhaps the most important being the ratio of antigen to antibody in circulation (Weigle, 1958). Most of the immune complexes formed in circulation that are of a size greater than 11S ($>Ag_2Ab_2$) are rapidly cleared by the mononuclear phagocyte system, in particular by the Kupffer cells of the liver (Benacerraf *et al.*, 1959; Arend & Mannik, 1971). Smaller immune complexes, formed in antigen excess, have been found to remain longer in circulation, and some formed with low-mol.-wt antigens or haptens were found to circulate for considerable periods (Schmidt *et al.*, 1974).

The reticuloendothelial system can be saturated by large quantities of circulating soluble immune complexes (Haakenstad & Mannik, 1974). Immune complexes containing tumour antigen have been found in the sera of tumour-bearing animals (Thomson *et al.*, 1973) and the presence of such complexes has been adduced from the finding that tumour-bearer sera can abrogate lymphocyte cytotoxicity (Sjögren *et al.*, 1971; Baldwin *et al.*, 1972). These observations have led to the concept that impairment of the immune clearance system in animals bearing tumours may be responsible for the continued presence of circulating immune complexes.

To investigate the immune clearance system in normal and tumour-bearing animals we have followed the disappearance from circulation of complexes formed *in vivo* between radiolabelled human serum albumin (HSA) and specific antibody. We have monitored the clearance of the immune complexes from circulation, both as the disappearance of labelled antigen and as the decrease in radioactivity that was precipitable from plasma by 2·5% polyethylene glycol.

### MATERIALS AND METHODS

### *Immunization procedure*

Eight adult Lister Hooded/Cbi rats each weighing ~300 g were immunized with 200 μg alum-precipitated human serum albumin (HSA) (AB Kabi, Stockholm, Sweden) per rat in 2 separate sites and on 2 occasions separated by an interval of 3 weeks.

### In vivo *clearance of $^{125}$I-HSA*

*Normal animals.*—Two weeks after the second immunization, the rats were anaesthetized with ether and 360 μg radiolabelled HSA in 0·4 ml isotonic saline was injected *via* the sublingual vein. Blood samples of about

* Present address: Department of Chemical Pathology, Guy's Hospital Medical School, London.

0·2 g were taken from the tail into weighed tubes containing 0·1 ml of 0·2M EDTA (pH 7·5) and the total radioactivity was assayed in a Packard Auto-gamma spectrometer. An additional blood sample was taken to determine the haematocrit value. From these data the radioactivity per gram of blood was calculated, and the rate of clearance determined using the equation:

$$\% \text{ injected } ^{125}\text{I-HSA in circulation} = \frac{C_b \times V_b}{C_i} \times 100$$

Where at time t: $C_b$ is the ct/min $^{125}$I/g blood, $V_b$ is the animal's blood volume in ml (estimated as 7% of the body wt) and $C_i$ is the ct/min $^{125}$I-HSA injected at zero time.

The blood sample was centrifuged and the radioactivity in 0·1 ml of the plasma-EDTA supernatant was determined. The rate of plasma clearance was calculated from the equation:

$$\% \text{ injected } ^{125}\text{I-HSA in plasma} = \frac{(W_b \times H + 0{\cdot}1) \times C_p \times V_b}{W_b \times C_i} \times 100$$

Where at time *t*: $W_b$ is the weight of the blood sample, H is the haematocrit value, and $C_p$ is the ct/min $^{125}$I/ml plasma-EDTA. ($V_b$ and $C_i$ as above.)

To each 0·1ml sample of plasma-EDTA was added 1·0 ml of a solution containing 2·5% (w/v) polyethylene glycol 6000 (PEG, British Drug Houses Ltd, Poole, Dorset) and 0·075M NaCl in 0·1M borate buffer (pH 8·3) (Zubler *et al.*, 1976). After 2 h at 0°C the tubes were centrifuged at 1500 *g* for 45 min in an MSE Mistral 6L centrifuge at 4°C. The supernatants were discarded and the radioactivity remaining in the tubes determined. The results were expressed as:

(1) % plasma $^{125}$I-HSA that was precipitable by PEG;

(2) % injected $^{125}$I that was precipitable by PEG.

*Tumour-bearing animals.*—The clearance of $^{125}$I-HSA from 4 normal and 4 immune rats was examined 10 days after these animals had been injected i.v. with $2 \times 10^4$ cells of the Hooded rat leukaemia (HRL; Wrathmell, 1976). The passage number and cell concentration used caused death of the rats within 14 days, though they remained ostensibly well until 48 h before death. The experimental protocol was the same as that described for the non-tumour-bearing rats.

*Labelling of human serum albumin with $^{125}$Iodine*

HSA (AB Kabi, Stockholm, Sweden) was labelled with $^{125}$Iodine by the chloramine T method (McConahey & Dixon, 1966) to sp. act. 0·8–1·0 μCi/μg. Before use, the $^{125}$I-HSA was diluted 10-fold with unlabelled HSA to give a total input of 360 μg/rat.

*Estimation of plasma levels of rat anti-HSA*

These were determined by passive haemagglutination, using HSA coupled to sheep red blood cells as the antigen source.

RESULTS

The clearance of $^{125}$I-HSA from the circulation of normal and immunized Hooded rats was followed for 6 h and the results are shown in the Figure. $^{125}$I-HSA was rapidly cleared from the circulation of immune rats, whereas in control animals the level of radioactivity in the blood

FIG.—Clearance of $^{125}$I-HSA from the circulation of normal (●, ○) or leukaemic (▲, △) Hooded rats that had been pre-immunized with HSA (open symbols) or were non-immune (closed symbols).

TABLE I.—*Clearance of $^{125}$I-HSA from the circulation of HSA-immune and non-immune Hooded rats*

| Time (min) | % injected $^{125}$I* In circulation Control | Immune | In plasma Control | Immune | Precipitable from plasma by 2·5% PEG Control | Immune |
|---|---|---|---|---|---|---|
| 10 | 89·3±1·4 | 30·0±12·8 | 89·3±1·4 | 4·6±2·0 | 1·1±0·23 | 3·0±1·77 |
| 20 | 86·6±5·3 | 10·8±4·8 | 86·0±6·1 | 4·5±1·9 | 1·1±0·10 | 3·0±1·45 |
| 30 | 83·5±8·3 | 5·7±1·6 | 83·5±8·3 | 3·4±1·0 | 0·7±0·04 | 1·5±0·71 |
| 60 | 77·2±6·8 | 7·4±1·0 | 73·7±1·9 | 3·0±0·4 | 0·7±0·04 | 0·4±0·08 |
| 90 | 75·5±6·6 | 7·1±1·0 | 68·6±5·0 | 3·4±0·7 | 0·7±0·09 | 0·3±0·05 |
| 120 | 69·5±3·6 | 8·4±1·8 | 66·2±2·9 | 4·2±1·1 | 0·7±0·09 | 0·2±0·04 |
| 240 | 57·8±5·4 | 8·2±0·7 | 49·2±3·7 | 4·1±0·5 | 0·7±0·05 | 0·2±0·03 |
| 360 | 49·6±2·7 | 8·1±0·8 | 42·9±3·1 | 4·0±0·4 | 0·5±0·05 | 0·2±0·07 |

* Results are the means ±s.d. for 4 HSA-immune or 4 control animals.

TABLE II.—*Clearance of $^{125}$I-HSA from the circulation of HSA-immune and non-immune HRL tumour-bearing rats.*

| Time (min) | % injected $^{125}$I* In circulation Control | Immune | In plasma Control | Immune | Precipitable from plasma by 2·5% PEG Control | Immune |
|---|---|---|---|---|---|---|
| 10 | 90·7±6·0 | 26·6±18·0 | 89·8±7·4 | 17·0±23·8 | 0·8±0·21 | 4·2±3·65 |
| 20 | 95·8±6·0 | 14·5±11·5 | 95·0±1·7 | 11·5±14·0 | 0·9±0·29 | 2·6±1·52 |
| 30 | 90·3±3·8 | 12·2±9·9 | 89·3±2·5 | 10·0±11·9 | 0·9±0·20 | 1·3±0·53 |
| 60 | 85·2±4·4 | 11·4±4·6 | 84·7±4·8 | 6·1±3·6 | 0·8±0·17 | 0·3±0·07 |
| 90 | 75·7±5·7 | 10·1±3·4 | 73·6±3·1 | 5·0±1·3 | 0·7±0·22 | 0·3±0·22 |
| 120 | 75·7±7·4 | 10·0±2·9 | 75·6±7·3 | 4·8±0·8 | 0·8±0·21 | 0·2±0·12 |
| 240 | 62·9±3·3 | 9·3±2·9 | 61·9±2·1 | 4·8±0·2 | 0·5±0·11 | 0·1±0·03 |
| 360 | 59·2±8·7 | 8·0±2·7 | 56·2±7·9 | 4·0±0·4 | 0·6±0·33 | 0·1±0·02 |

* Results are the means ± s.d. for 4 HSA-immune or 4 control animals.

decreased slowly and exponentially so that 6 h after injection about half the injected dose remained in circulation. During this time, virtually all the circulating $^{125}$I-HSA in the controls was present in the plasma fraction of the blood samples (Table I). In the immune rats, where at 10 min 30% of the $^{125}$I-HSA remained in circulation, only 4·6% of the input was found in the plasma fraction, leaving 25·4% associated with the blood cells. These results show that soluble complexes of $^{125}$I-HSA–rat anti-HSA formed i.v. were rapidly taken up by the cellular components of the blood and cleared from circulation.

Samples of plasma were assayed at intervals for the presence of $^{125}$I-HSA–rat anti-HSA complexes that were precipitable by PEG at a final concentration of 2·5% (Tables I and III). PEG-precipitable soluble immune complexes were cleared from circulation within 1 h though some PEG-precipitable material could be detected in the plasma of immune rats throughout the experiment (Table III).

TABLE III—*% of $^{125}$Iodine remaining in the plasma of normal or leukaemic rats that was precipitable by 2·5% PEG*

| Time (min) | Non-tumour-bearing* Control | HSA-immune | Tumour-bearing* Control | HSA-immune |
|---|---|---|---|---|
| 10 | 1·2±0·2 | 57·4±19·2 | 0·9±0·2 | 48·9±25·7 |
| 20 | 1·2±0·3 | 65·1±5·2 | 0·9±0·3 | 39·3±20·7 |
| 30 | 0·8±0·2 | 45·5±7·1 | 1·0±0·3 | 24·3±14·5 |
| 66 | 0·9±0·1 | 13·6±2·7 | 0·9±0·2 | 6·4±3·4 |
| 90 | 1·0±0·1 | 8·0±3·4 | 1·0±0·2 | 6·7±4·5 |
| 120 | 1·0±0·3 | 5·7±2·1 | 1·0±0·2 | 4·3±1·0 |
| 240 | 1·4±0·1 | 3·7±0·4 | 0·8±0·2 | 1·4±0·5 |
| 360 | 1·0±0·2 | 4·1±1·5 | 1·0±0·4 | 1·1±0·5 |

* Results are the means ± s.d. for 4 HSA-immune or 4 control animals.

To discover whether tumour-bearing animals retained their ability to clear soluble immune complexes from circulation, the experiment was repeated using HSA-immune and control rats that had been given $2 \times 10^4$ HRL cells i.v. 10 days before (see Methods). This Hooded rat leukaemia is a thy 1.1-bearing leukaemia (Wrathmell, 1976) without Fc receptors. The results presented in the Figure and Table II show that the clearance of $^{125}$I-HSA from the circulation in both immune and control tumour-bearing rats was not significantly different from that of non-tumour-bearing animals. Again, $^{125}$I-HSA complexes could be detected in the plasma of tumour-bearing immune rats by precipitation with 2·5% PEG (Table III). In one of the HSA-immune tumour-bearing animals, about 8% of the input of $^{125}$I-HSA was found to be precipitable by PEG from plasma taken at 10 min but in this animal no cell-bound radioactivity was detected then or subsequently. In the other tumour-bearing immune animals, however, more than 70% of the $^{125}$I-HSA in circulation at 10 min was cell-bound.

The haemagglutination titres of anti-HSA in all sera taken at the start of each experiment lay between 1/3200 to 1/6400 in the immune rats, whilst control rats were negative.

### DISCUSSION

After a single i.v. infusion of $^{125}$I-HSA, the immune complexes formed *in vivo* by HSA-immune rats were cleared rapidly from circulation. We have shown that a proportion of these complexes were soluble and could be detected *in vitro* by precipitation from plasma with PEG. However, the bulk of the radiolabelled antigen in the circulation of immune rats was bound to blood cells during the initial phase of immune clearance. In the control animals $^{125}$I-HSA was cleared more slowly, all of the radioactivity being in the plasma fraction and none precipitable by PEG.

The reticuloendothelial system can be saturated by injecting immune complexes in slight antibody excess into normal mice (Haakenstad & Mannik, 1974). To avoid this complication an antigen dosage was used that did not saturate the system and did allow normal clearance. Under these conditions HSA-immune, leukaemia-bearing Hooded rats given the same quantity of antigen as control HSA-immune animals were capable of clearing the immune complexes formed, and there was no significant difference in either the rate or extent of clearance. Again, a large proportion of the $^{125}$I-HSA remaining in the circulation of immune animals was found to be bound to blood cells. The Hooded rat leukaemia does not bear Fc receptors so that it is unlikely that HRL cells in the peripheral blood assisted in the clearance of immune complexes. We conclude that the presence of the rat leukaemia did not affect the normal mechanism for clearance of the HSA-immune complexes.

The finding that most of the HSA-immune complexes in circulation were cell-bound leads us to question the validity of some methods currently used for monitoring immune complexes *in vivo*, which rely on the use of serum or plasma alone. Experiments are in progress to determine the peripheral blood cells involved in the clearance of immune complexes.

This work was supported by grants from the Medical Research Council and the Cancer Research Campaign. One of us (P.S.S.) was the recipient of a Gordon Jacobs Research Fellowship.

### REFERENCES

AREND, W. P. & MANNIK, M. (1971) Studies on antigen–antibody complexes. II: Quantification of tissue uptake of soluble complexes in normal and complement depleted rabbits. *J. Immunol.*, **107**, 63.

BALDWIN, R. M., PRICE, M. R. & ROBINS, R. A. (1972) Blocking of lymphocyte-mediated cytotoxicity for rat hepatoma cells by tumour-specific antigen complexes. *Nature* (*New Biol.*), **238**, 185.

BENACERRAF, B., SEBESTYEN, M. & COOPER, N. S. (1959) The clearance of antigen–antibody complexes from the blood by the reticuloendothelial system. *J. Immunol.*, **82**, 131.

DIXON, F. J. & TALMAGE, D. W. (1951) Catabolism of $^{131}$I-labelled bovine gamma globulin in immune and non-immune rabbits. *Proc. Soc. Exp. Biol. Med.*, **78**, 123.

HAAKENSTAD, A. O. & MANNIK, M. (1974) Saturation of the reticuloendothelial system with soluble immune complexes. *J. Immunol.*, **112**, 1939.

McCONAHEY, P. J. & DIXON, F. J. (1966) A method of trace iodination of proteins for immunologic studies. *Int. Arch. Allergy Appl. Immunol.*, **29**, 185.

SCHMIDT, D. H., KAUFMAN, B. M. & BUTLER, V. P., JR (1974) Persistence of hapten–antibody complexes in the circulation of immunised animals after a single intravenous injection of hapten. *J. Exp. Med.*, **139**, 278.

SJÖGREN, H. O., HELLSTRÖM, I., BANSAL, S. C. & HELLSTRÖM, K. E. (1971) Suggestive evidence that the blocking antibodies in tumour-bearing individuals may be antigen–antibody complexes. *Proc. Natl Acad. Sci. U.S.A.*, **64**, 161.

THOMSON, D. M. P., ECCLES, S. & ALEXANDER, P. (1973) Antibodies and soluble tumour-specific antigens in blood and lymph of rats with chemically induced sarcomata. *Br. J. Cancer*, **28**, 6.

WEIGLE, W. O. (1958) Elimination of antigen–antibody complexes from sera of rabbits. *J. Immunol.*, **81**, 204.

WRATHMELL, A. B. (1976) The growth patterns of two transplantable acute leukaemias of spontaneous origin in rats. *Br. J. Cancer*, **33**, 172.

ZUBLER, R. H., LANGE, G., LAMBERT, P. H. & MESCHER, P. A. (1976) Detection of immune complexes in unheated sera by a modified $^{125}$IClq binding test. *J. Immunol.*, **116**, 232.